# A Fluorometric Method for the Quantification of Cell Number in Complex Differentiating Osteoblast-Osteocyte Cultures

**DOI:** 10.3390/mps1020014

**Published:** 2018-04-12

**Authors:** Dongqing Yang, Asiri R. Wijenayaka, Gerald J. Atkins

**Affiliations:** Biomedical Orthopaedic Research Group, Centre for Orthopaedic and Trauma Research, The University of Adelaide, 5000 Adelaide, SA, Australia; asiri.wijenayaka@adelaide.edu.au (A.R.W.); gerald.atkins@adelaide.edu.au (G.J.A.)

**Keywords:** osteoblast, osteocyte, GelRed™, mineralisation, cell number, fluorometric quantification

## Abstract

Osteoblast/osteocyte cultures continue to emerge as essential tools for bone biology research in vitro. The change in cell number is an important parameter to be considered for investigating osteogenic differentiation. However, there is no reliable method for quantifying absolute cell count in differentiating osteoblast/osteocyte cultures because of their strongly adherent, multi-layered, super-confluent nature, and their accumulated extracellular matrix production which progressively mineralises in vitro. We developed a practical, simple and cost-effective method based on the fluorometric quantification of a nucleic dye, GelRed™, to enumerate cell number in osteoblast/osteocyte cultures. This method may also be suitable for quantifying cell numbers on other mammalian adherent cell types.

## 1. Introduction

Quantification of cell number in mammalian adherent cell types in vitro, particularly osteoblast/osteocyte differentiation cultures, is crucial and yet challenging for many biological studies. Deficiencies of currently available techniques are summarised as follows: (a) suspension cell counting is not preferable when cultures need to be used for other tests and is associated with error due to the difficulty in generating single cell suspensions; (b) image-based cell counting is not practical for cells with super-confluency where the visualisation of individual cells is impaired; (c) assays including 3-(4,5-Dimethylthiazol-2-yl)-2,5-Diphenyltetrazolium Bromide (MTT) and crystal violet staining, which measure either mitochondrial activities or total protein levels as a correlation with viable cell number, become inaccurate if cell metabolism changes with time point(s)/treatment(s) [1]; and (d) genomic DNA content measurement by the most commonly used fluorometric detecting reagent, PicoGreen^®^ (Life Technologies, Grand Island, NY, USA), may not be compatible if cells are engineered with green fluorescent protein (GFP) [2], or, in the case of osteoblast/osteocyte cultures, have deposited calcium phosphate mineral, which elicits green auto-fluorescence [3].

Differentiating osteoblast/osteocyte cultures feature heavily overlapping cells in either two-dimensional or 3D (three-dimensional) cultures and have large quantities of extracellular matrix and mineral deposition *in situ* [4]. In these cultures, strong green auto-fluorescence attributed to mineralisation has been reported [3]; in addition, either the generation of single cell suspension or visualisation of separated cells is almost impossible. In addition, in osteogenic cultures, various cellular activities, including energy metabolism, cytoskeleton, gene expression, have been demonstrated to undergo dramatic shifts during the transition of osteoblast to osteocyte [5]. To overcome these difficulties, we have optimised the fluorometric-based quantification of genomic DNA content to allow the accurate determination of cell number in such complex cultures, using a commercially available reagent, GelRed™ (Biotium, Fremont, CA, USA) Nucleic Acid stain.

## 2. Materials and Methods

### 2.1. Reagents and Equipment

Adherent cell line/primary cell of interest: e.g., mouse osteogenic cell lines MLO-A5 [6] and IDG-SW3 [2], or human primary bone-derived cells (NHBC) [7].

Osteogenic differentiation media: α-MEM (Cat. No. 12561, Life Technologies) containing: 10% v/v foetal bovine serum (Thermo-Fisher Scientific, Scoresby, VIC, Australia); tissue culture additives including 100 IU/mL penicillin/streptomycin and 2 mM l-glutamine (Life Technologies); β-glycerophosphate disodium salt hydrate (Sigma-Aldrich, St. Louis, MO, USA) at final concentrations of 1.8 mM for NHBC, 4 mM for IDG-SW3 and 10 mM for MLO-A5; and Ascorbate-2-phosphate at final concentration of 100 µM (Sigma-Aldrich) (Note: For culturing alternative cell types, appropriate media should be used).

Fixative: Formalin solution, neutral buffered, 10% (10% NBF) (Sigma-Aldrich).

Nucleic acid staining solution: Dilute the commercial stock of GelRed™ (10,000×) 2× in water as the staining solution. (The properties of GelRed™ are summarised as below: (1) It is designed for visualising double stranded DNA in agarose gel with the sensitivity of detecting 500 pg DNA. (2) It is non-toxic and non-mutagenic because it does not bind DNA in living cells due to its membrane impermeability. In our experience, this dye can be added to live cultures up to 72 h with no evidence of toxicity (data not shown) (3) Three optimal excitation wavelengths, 290 nm, 355 nm and 544 nm, can be used to achieve the same emission of 600 nm. Such fluoro-characterisation does not interfere with the commonly used GFP/FITC (fluorescein isothiocyanate) or RGP (red fluorescent protein)/TRITC (tetramethylrhodamine-isothiocyanate) channels.)

Digestion solution: Dissolve Proteinase K powder (Sigma-Aldrich) in Milli-Q water and adjust the concentration to 20 ng/mL.

Equipment: Nunc™ Cell-Culture Treated Multi-dishes, 24-well (Thermo-Fisher Scientific); microplates for fluorescence-based Assays, 96-well, black colour wall (Thermo-Fisher Scientific); FLUOstar OPTIMA Microplate Reader (BMG Labtech, Ortenberg, Germany); and 5% CO_2_/37 °C humidified cell culture incubator.

### 2.2. Experimental Procedure

In this study, the osteogenic cell line MLO-A5 [6] was chosen as an example to demonstrate the application of this cell quantification method to different forms of cell culture: suspension and adherent mono-layer culture without mineralisation. Well characterized NHBC cultures [7] were utilized to validate the assay reliability on high-confluency multi-layer culture with mineralisation. The osteogenic cell line IDG-SW3 [2] was employed to enumerate cell number over a three-week differentiation period associated with the complexity of accumulating transgenic GFP expression during this period.

#### 2.2.1. Sample Preparation

**Figure 1:**
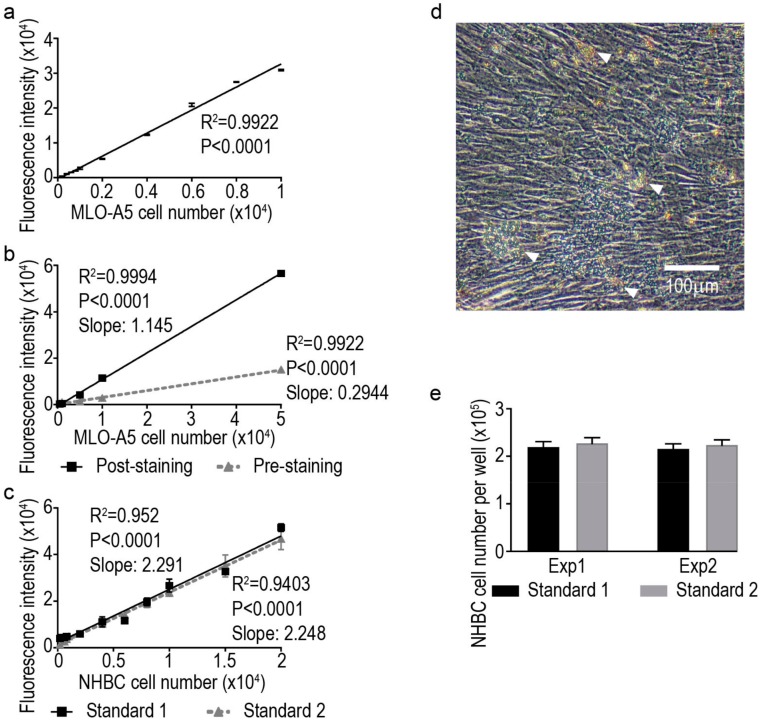
(**a**) Standard curve generated from MLO-A5 suspension cultures; (**b**) standard curves generated from MLO-A5 sub-confluent monolayer cultures using post-staining (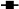) and pre-staining (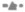) methods; (**c**) standard curves generated from human primary bone-derived cells (NHBC) suspension cultures from two independent experiments; (**d**) representative image of a differentiated NHBC culture under phase-contrast with high level of confluence and deposited mineral nodules (indicated by arrowheads); and (**e**) cell number measurements from two independent experiments by returned calculation using the two standard curves above, respectively. Each bar graph represents the average cell number from four independent culture wells and the error bar represents the standard error of the mean.

**Figure 2:**
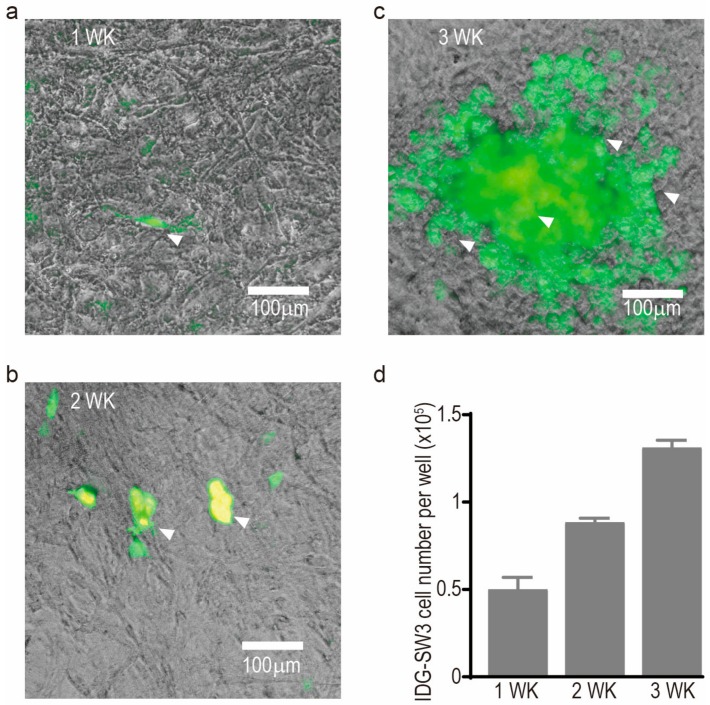
Representative images of IDG-SW3 cultures at: one week (**a**); two weeks (**b**); and three weeks (**c**) of osteogenic differentiation, with arrowheads indicating the increasing amount of green fluorescent protein (GFP) production; cell number measurement at each of the time points (**d**). Each bar graph represents the average cell number from four independent culture wells and the error bar represents the standard error of the mean.

Similar to other methods, this assay relies on the linear regression principle and requires a standard as reference when calculating actual cell numbers in experimental samples. Here, we generated standards using suspension cultures titrated by serial dilutions: from 10^4^ to 2 × 10^2^ cells for MLO-A5 and from 2 × 10^4^ to 2 × 10^2^ cells for NHBC (Figure 1a, c). Test samples from cell monolayers were prepared by adding MLO-A5 cells (500–50,000 cells) to 24-well tissue culture plates, allowing time for complete cell attachment (2 h at 37 °C) before proceeding to the next step (Figure 1b). For testing cell number in conditions of high confluence and extensive mineralisation, NHBC and IDG-SW3 cells were seeded at a density of 10^4^ cells/well in 24-well plates and differentiated for 28 and 21 days, respectively. A considerable amount of mineral and extracellular matrix was deposited in both cultures and the accumulating expression of GFP was also observed in IDG-SW3 cultures (Figure 1d and Figure 2a–c). (Note: For referencing monolayer cell cultures, the utilisation of the same cell type for the suspension standard is valid, as this method measures the total genomic DNA content, regardless of their morphology or adherence to a surface. However, the use of a single standard to reference multiple cell types is not recommended.)

#### 2.2.2. Stabilization of Cells

Fix cells in the form of either suspension or monolayer culture by 10% NBF for 30 min at room temperature. Use the volume of 1 mL for fixing 1 × 10^6^ cells in suspension or 250 µL for covering one 24-well. Then, remove residual fixative by 1× PBS wash of cells twice. For the removal of liquid in monolayer cultures, directly aspiration is preferred while pelleting cells by centrifugation at 200 g for 5 min is required for cells in suspension. (Note: If other tests on live cultures will be carried out on the same experimental samples, this assay should be performed as the last step. The appropriate fixative should be selected to ensure its compatibility with other assay(s), i.e., non-formaldehyde-based fixative should be used in combination with nuclear hybridisation in situ; non-acidic fixative should be chosen if alkaline phosphatase activity is to be measured.)

#### 2.2.3. Generation of Cell Lysates

Homogenize cells in digestion solution (dissolve proteinase K power in water and adjust the concentration to 20 ng/mL) in a 5% CO_2_/37 °C humidified incubator for 4 h. For suspension cultures, vigorous agitation using a laboratory mixer is required to generate homogenous lysates; repetitive drawing of the liquid is required for monolayer cultures. (Note: According to the product information, this proteinase K product is optimised for activity at 65 °C. However, in this case, the final lysate volume is critical for measurement, we recommend the digestion conditions of 37 °C and a humidified environment to minimise evaporation. The digestion procedure can be improved by increasing proteinase K concentration and/or incubation time. For different cell types, optimisation of digestion is recommended.)

#### 2.2.4. GelRed^TM^ Staining on Genomic DNA

Mix equal volumes of cell lysate and GelRed™ staining solution (2×) in wall-insulated 96-well plates and the samples are ready for measuring fluorescent intensity in seconds. For the determination of assay background, include samples with 1× GelRed™ solution in water. (Note: In the format of 96-wells, the combination of 50 µL lysate and 50 µL staining solution has been tested as optimal and titration of volume for other plate formats is recommended. A black colour wall-insulated 96-well plate is suggested for minimising fluorescence interference between neighbouring wells. This method is compatible for particular applications where pre-staining of GelRed™ on fixed cells prior to digestion is required. However, the sensitivity of the assay is lower with pre-staining in comparison to post-digestion staining, evidenced by the lower slope constant of the linear regression (Figure 1b).

#### 2.2.5. Fluorescence Intensity Reading and Cell Number Determination

Perform fluorescence intensity reading of cell lysate/GelRed™ mixtures using a FLUOstar OPTIMA Microplate Reader, or similar instrument, with the adjustments of excitation at 355 nm/emission collection at 600 nm. Excitation at 544 nm was also tested and identical results were achieved, in terms of final cell number and assay quality (data not shown). The values (with background subtracted) of fluorescence intensity of test samples can be returned to standard and calculated using the standard curve (regression) equation to determine the actual cell number. (Note: As all fluorescent plate readers have their own laser gain adjustment mechanism, such adjustment is usually determined from the sample with the highest recorded fluorescence. For achieving inter-assay consistency, it is important that the device-specific gain level is used. If the fluorescence reading is out of the range of the standard curve, appropriate dilution of samples in water should be performed to ensure the measurement falls in standard curve reading. In bar graphs in Figure 1e, all samples were diluted 10-fold before proceeding to the final reading step.)

## 3. Results

In our experimental setting, the quantification of cell number in both suspension and monolayer forms of cultures fitted by linear regression with very high reliability in all three tested cell types (Figure 1a–c; data not shown for IDG-SW3). For NHBC, the generation of standard curves were carried out twice independently and a high degree of reproducibility was achieved (Figure 1c). For the end-point measurement of four-week differentiated NHBC cultures, which characteristically have a considerable degree of mineral deposition and extracellular matrix production (Figure 1d), two independent experiments were performed and the two-separate sets of GelRed™ fluorometric measurements were returned to both standard curve equations to calculate the cell number. Almost identical cell numbers per well were obtained by the enumeration of both experiments, using either of the standard equations for calculation (Figure 1e). These data validated the intra- and inter-assay reproducibility of this approach.

The osteoblastic cell line IDG-SW3 [2] was employed in this study as an example of a cell line expressing GFP, a commonly used cellular marker for labelling cell component(s). Originally, this cell line was engineered to express GFP under control of the dentin matrix acidic phosphoprotein (*Dmp1*) promoter, the activity of which increases during differentiation to a mature osteocyte stage. Under our osteogenic culture conditions, the expected time-dependent accumulation of GFP was observed (Figure 2a–c). For the purpose of fluorometric quantification of genomic content, the utilisation of the popular reagent, PicoGreen^®^, or any other fluorescent reagent that is excited under GFP/FITC channel, should be avoided in this case as this dye shares the same excitation channel with GFP. Here, we demonstrate that the application of GelRed™ for fluorometric enumeration of cell number offers very high reproducibility in such samples (Figure 2d). In addition, the lack of interaction between GelRed™ and GFP was confirmed by the zero-background reading when GFP-containing cell lysate was excited at the GelRed™ fluorescence channel (data not shown).

## 4. Discussion

In this study, we demonstrate a cost-effective and simple method for the determination of cell number in mammalian cultures. The advantage of this protocol is the ability to accurately assess cell number without the bias contributed by changes in metabolism (mitochondrial activity) and/or cell morphology (total protein content) governed by the consistency of the amount of genomic DNA in each mammalian cell. Our data demonstrate very high quality of linear regression fit in both suspension and sub-confluent monolayer cultures, by either mouse cell line or human primary cell types. Using this method, high reproducibility between independent experiments is achievable when aiming to quantify cell number in super-confluent/heavily mineralised cultures. This protocol is also valuable for investigating cell number change during the process of osteogenic differentiation, in which cell morphology changes significantly from early to late time points. This approach is potentially compatible with 3D culture, however, the efficiency of cell homogenisation from different 3D structures would need to be determined in each case. The application of this method for counting cell number in bacterial assays should be cautious, as bacteria generated biofilm is a nucleic acid enriched material, which might compromise the assay specificity.
